# Nutraceuticals as Potential Therapeutic Modulators in Immunometabolism

**DOI:** 10.3390/nu15020411

**Published:** 2023-01-13

**Authors:** Gonzalo Alba, Hala Dakhaoui, Consuelo Santa-Maria, Francisca Palomares, Marta Cejudo-Guillen, Isabel Geniz, Francisco Sobrino, Sergio Montserrat-de la Paz, Soledad Lopez-Enriquez

**Affiliations:** 1 Department of Medical Biochemistry and Molecular Biology, and Immunology, School of Medicine, University of Seville. Av. Sanchez Pizjuan s/n, 41009 Seville, Spain; 2 Department of Biochemistry and Molecular Biology, School of Pharmacy, University of Seville, 41012 Seville, Spain; 3 Department of Pharmacology, Pediatry, and Radiology, School of Medicine, University of Seville. Av. Sanchez Pizjuan s/n, 41009 Seville, Spain; 4 Distrito Sanitario Seville Norte y Aljarafe, Servicio Andaluz de Salud, 41008 Seville, Spain

**Keywords:** tolerogenic dendritic cells, macrophages, regulatory T cells, regulatory B cells, proteosome, autophagy, succinate, glycolysis, oxidative phosphorylation, tricarboxylic acid cycle

## Abstract

Nutraceuticals act as cellular and functional modulators, contributing to the homeostasis of physiological processes. In an inflammatory microenvironment, these functional foods can interact with the immune system by modulating or balancing the exacerbated proinflammatory response. In this process, immune cells, such as antigen-presenting cells (APCs), identify danger signals and, after interacting with T lymphocytes, induce a specific effector response. Moreover, this conditions their change of state with phenotypical and functional modifications from the resting state to the activated and effector state, supposing an increase in their energy requirements that affect their intracellular metabolism, with each immune cell showing a unique metabolic signature. Thus, nutraceuticals, such as polyphenols, vitamins, fatty acids, and sulforaphane, represent an active option to use therapeutically for health or the prevention of different pathologies, including obesity, metabolic syndrome, and diabetes. To regulate the inflammation associated with these pathologies, intervention in metabolic pathways through the modulation of metabolic energy with nutraceuticals is an attractive strategy that allows inducing important changes in cellular properties. Thus, we provide an overview of the link between metabolism, immune function, and nutraceuticals in chronic inflammatory processes associated with obesity and diabetes, paying particular attention to nutritional effects on APC and T cell immunometabolism, as well as the mechanisms required in the change in energetic pathways involved after their activation.

## 1. Introduction

The term “nutraceutical” was proposed by DeFelice in 1984 as any substance that can be a food or part of a food that provides medical or health benefits, including the prevention and treatment of disease [1]. Other authors have amplified or redefined this concept as a mix of substances able to interact with individual DNA molecules as a function of the environment [2,3]. Moreover, nutraceuticals can perform as cellular and functional modulators, contributing to the homeostasis of physiological processes [3]. Therefore, in an inflammatory microenvironment, these functional foods can act on the immune system by modulating or balancing the exacerbated proinflammatory response. Because of polydrug approaches that are often used in these processes, their management can exhibit adverse side effects; therefore, the nutraceutical capacity represents an active option to use therapeutically for health or the prevention of pathologies, such as cancer, gastroenterological disorders, inflammatory and neurodegenerative diseases, and infections [4,5,6,7].

The main function of the immune system is defense against foreign and/or malignant cells through nonspecific or specific mechanisms that induce innate and adaptative immune responses, respectively. For this, professional antigen-presenting cells (APCs), such as dendritic cells (DCs), macrophages, and B lymphocytes, after identifying external or internal danger signals, i.e., pathogen-associated molecular patterns (PAMPs) or danger-associated molecular patterns (DAMPs), acquire an activated state [8]. Moreover, during this process, APCs migrate to lymphatic nodes to interact with T lymphocytes and induce a specific effector response [9], with activation of intracellular signaling cascades, leading to the induction of a general proinflammatory response [8]. This change of state, which includes phenotypical and functional modifications from the resting state to the activated and effector state from these immune cells, supposes an increase in their energy requirements that affect their intracellular metabolism [10]. This implies deep changes in different metabolic pathways, such as glycolysis, the tricarboxylic acid (TCA) or Krebs cycle, the pentose phosphate pathway (PPP), fatty acid (FA) oxidation (FAO), fatty acid synthesis (FAS), amino acid metabolism, and oxidative phosphorylation (OXPHOS) [10,11].

For this, leukocytes are subjected to continuous metabolic reprogramming, not only as a consequence of oxygen and nutrient levels, but also driven by microenvironmental signals, such as PAMPs and DAMPs, through various signaling pathways involved in activation, cell differentiation, and/or proliferation [12,13,14,15]. Furthermore, metabolic signaling drives cell fate [15,16].

**Figure 1:**
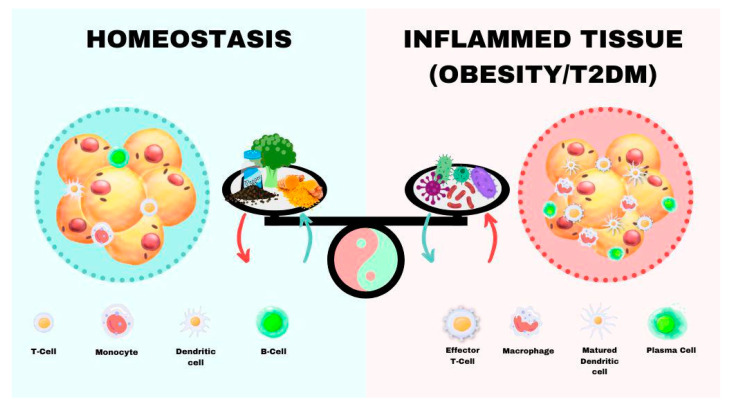
Factors that affect and/or regulate immunometabolism in the tissue microenvironment.

The term “immunometabolism” is considered an “interface” of the immune system and metabolism [17]. Currently, the scientific community speaks openly about how an imbalance in signaling pathways in immunometabolism can lead to the pathogenesis of metabolic disorders, such as obesity, diabetes, metabolic syndrome hypertension, cancer, and autoimmune diseases [18,19,20,21,22]. Indeed, the scientific community is conducting an active search for the possible mechanisms involved in intracellular metabolism that allow the regulation of immune responses, seeking a balance between the functions of effector and regulatory immune cells. The inflammatory process plays an essential role in the promotion of metabolic abnormalities that cause diseases such as obesity and type 2 diabetes mellitus (T2DM), and metabolic factors, in turn, regulate immune cell functions. Hence, obesity as the main inducer of a local or systemic chronic inflammation is a main inductor for T2DM [23]. High blood glucose levels and the related inflammation can generate angiopathies in the circulatory system. Moreover, the impaired innate and adaptative systems, together with metabolic dysregulation, also raises the sensitivity of patients to severe acute respiratory syndrome coronavirus 2 (SARS-CoV-2) [24]. Furthermore, an interaction between the unbalanced metabolic state and these low-grade chronic inflammatory responses can lead, through a vicious cycle, to the development of metabolic diseases, such as T2DM (Figure 1). Moreover, in the inflammatory process, autophagy is a fundamental biological process contributing to immunometabolism [25,26]. Thus, upon leukocytes identifying proinflammatory danger signals, autophagy is activated and initiates a protective cytoplasmic function, removing aggregated or damaged cytosolic proteins and damaged lysosomal membranes, such as DAMPs, inflammasome components, and type I interferon (IFN) regulators, through the multistage lysosome–homeostatic response termed “membrane repair, removal, and replacement” (MERiT). In this process, lysosomal damage inhibits the mechanistic target of rapamycin (mTOR) signaling pathway and activates AMP-activated protein kinase (AMPK) [26]. mTOR is a protein kinase regulator of intracellular metabolism, present in two signaling complexes, mTORC1 and mTORC2, which are composed of distinct protein-binding partners with different functions in the development, homeostasis, and differentiation of immune cells [27]. Moreover, AMPK is often associated with immunometabolic states compatible with anti-inflammatory activities and quiescence [26]. In this sense, at the removal phase, it is activated and inhibited by the mTOR signaling pathway. As mentioned before, AMPK acts in situations in which autophagy is activated. An increase in mTOR activity is related to inflammatory states and a greater response of macrophages and effector T cells, while AMPK is related to anti-inflammatory states [26,27].

Moreover, NOD-like receptors (NLRs) are types of PAMP and DAMP receptors (PRRs) with roles of sensing systemic and intracellular metabolic perturbations, particularly the NLRP3 inflammasome. NLRP3 acts in the maintenance of intracellular homeostasis and is activated by DAMPs, promoting innate and adaptive immune responses. NLRP3 activation is regulated through intracellular metabolic pathways, such as hexokinase-1-dependent glycolysis, especially within the myeloid linage [28]. However, other studies have reported that the disruption of glycolytic flux serves as an activating signal for NLRP3 [29,30].

Thus, metabolomic and pharmacometabolomic studies have achieved progress in the use of nutraceuticals, which can get modified during intestinal transit and metabolism. Furthermore, the gut microbiome also plays a vital role in the host’s response to any drug or nutrient [4].

The repolarization of immune cells toward a less inflamed phenotype by reprogramming metabolism using small molecules and metabolites may be reachable [31]. For this reason, to regulate the inflammation associated with these pathologies, intervention in metabolic pathways through the modulation of metabolic energy with nutraceuticals is an attractive strategy that allows inducing important changes in cellular properties that can redrive them toward a tolerogenic immunophenotype for inflammatory disease. Thus, we provide an overview of the link between metabolism, immune function, and nutraceuticals in chronic inflammatory processes associated with obesity and diabetes, paying particular attention to the nutritional effects on APCs and T cell immunometabolism, as well as the mechanisms involved in the change in energetic pathways involved after their activation.

## 2. Results

### 2.1. Nutrient-Sensing Pathways Affecting Immune Cell Development in Homeostasis

Immune homeostasis can be achieved when there is a balance between immunogenicity against non-self-antigens and tolerance to self [32]. In this sense, the immune system is regulated by the neuroendocrine axis, in which metabolites are key communication signals allowing a shift in the metabolic homeostasis of immune cells. This regulation is bidirectional; reciprocally, the immune system can modulate whole-body metabolism. Thus, immune cells, upon identifying danger signals, change their basal metabolic, phenotypic, and functional state to an activate state, thus modifying metabolic pathways and metabolite production so as to orchestrate an effector or regulator immune response. This effect was proposed by Warburg, similar to what is observed in cancer cells; when leukocytes are activated, glucose consumption rates promote metabolic changes, inducing *aerobic glycolysis* [33,34]. During glycolysis, glucose enters the cell through GLUT1 receptors to produce pyruvate and ATP, which enter the mitochondria to produce acetyl-CoA. Although glycolysis is an inefficient means of generating energy, it enables cells to redirect intermediates from the TCA cycle to generate essential metabolites that promote anabolic pathways that allow immune activation, such as an increase in the secretory machinery [22,30,35].

The energy requirements of immune cells when the body is in homeostasis vary depending on the immune cell type and the state in which the cells are based on the environment, being able to clearly differentiate among rest, anergy, or tolerance processes. In this review, to provide an understanding of the main pathways involved in immunometabolism, we focus on APCs and T cells, which represent the most studied cell subsets, due to the fact that they have great potential in the search for developing alternatives natural therapies for immunometabolic diseases, such as obesity and T2DM.

#### 2.1.1. APCs: Key Signaling Molecules Mediating Nutritional Effects on APCs

DCs, monocytes/macrophages, and B cells are well equipped to detect environmental cues and play dominant roles during homeostasis and inflammation in peripheral blood and injured tissue. DCs are a heterogeneous population of professional APCs that create a crucial link between the innate and adaptive immune responses, and they are the only cells that can interact with naive T lymphocytes. Nevertheless, immature and tolerogenic DCs have different metabolic profiles than immunogenic DCs (semi-mature and mature) [32]. Thus, resting or immature DCs, due to their characteristics as professional APCs, continuously take up molecules and release them into the environment if they are not immunogenic. This supposes continuous energy expenditure, showing catabolic metabolism for energy generation and cell maintenance. Thus, Zaccagnino et al. demonstrated active mitochondrial biogenesis during DC differentiation, suggesting a role of PGC-1α in mitochondrial biogenesis [36]. This metabolic state manifests active OXPHOS, which is associated with the longevity of quiescent homeostatic immune cells [30], driven by the TCA cycle fueled via FAO [37,38] and glutaminolysis, and it is largely regulated by AMPK [38]. In this sense, it was proposed that resting circulating monocytes switch to glycolysis when they take up molecules, whereas when alveolar macrophages phagocytose, they use OXPHOS [39].

B cells play a pleiotropic role in the immune system. In the resting state, B cells (naive B, memory B, and long-lived plasma cells) are naturally quiescent, although they have demonstrated differences; for example, memory B cells express a class-switched BCR in which the quiescent state may be imposed through different process [40]. In particular, resting naive B cells import relatively little glucose because they proliferate only minimally [41]. Moreover, SIRT1 is highly expressed in resting B cells [42]. Germinal center B cells in vivo also rely on glycolysis, although the precise metabolic basis for this requirement needs further exploration [43]. Anergic B cells are metabolically quiescent, with a light increase in glycolysis and oxygen consumption after lipopolysaccharide (LPS) stimulation in vitro and a tolerant environment influence on B cell metabolic reprogramming [44].

#### 2.1.2. T Cells: Key Signaling Molecules Mediating Nutritional Effects on T Cells

The immunometabolism of T cells has drawn interest mainly for their biological and therapeutic potential. Thus, the energy needs of lymphocytes depend on their state, and the immediate environment and aerobic glycolysis in lymphocytes may be important not only for cell proliferation but also for the differentiation of T cells into effector lymphocytes and for the production of effector cytokines [45].

Resting T cells require or generate low energy expenditure [46], mainly engaging mitochondrial metabolism to generate energy [47]. Thus, mTORC1 signaling is essential for T cell development in the thymus and homeostasis in the periphery; by contrast, mTORC2 activity is required for Th1 and Th2 cell differentiation, while it also regulates the migration of Tfh and Treg cells [27]. Moreover, it has been demonstrated that phosphoinositide 3-kinase/protein kinase B (PI3K/PKB, also called Akt) pathways and mTOR complexes also regulate Th17 differentiation both in vivo and in vitro [48]. Naive T lymphocytes resting in lymphatic nodes show a low cell division ratio, which uses low energy expenditure. They use metabolites that enter the TCA cycle, where ATP and reducing equivalents are generated, which subsequently increase the production of ATP upon entering the OXPHOS pathway [49]. When naive T lymphocytes are in a resting state, energy demands are met by glycolysis. This metabolic process is linked to antigen recognition in immune activation [22]. Precursor T cells need substantial energy as they are continuously migrating through the lymphatic system; they get this energy from FAO and the TCA cycle. Moreover, both resting CD4 and CD8 T cells use a predominantly oxidative metabolism. Thus, Nicoli et al. demonstrated that autophagy and the mTOR-dependent glycolytic pathway are key agents of antigen-driven priming in the naive CD8^+^ T cell pool, showing that naive CD8^+^ T cells also have lower concentrations of neutral lipids and fatty acid intake compared to memory CD8^+^ T cells. Contrary, cholesterol uptake was higher among naive CD8^+^ T cells compared to memory CD8^+^ T cells. Moreover, it was demonstrated that non-indispensable amino acids, such as l-carnitine, can promote the effector differentiation of naive CD8^+^ T cells [50].

Invariant natural killer T (iNKT) cells, the main category of natural killer T cells and a subpopulation of mature innate T lymphocytes [51], were identified as essential players in immunometabolism due to their capacity to respond to self-derived or microbially derived lipid antigens, such us α-galactosylceramide (α-GalCer), which is presented by CD1 molecules on APCs. Similar to these APCs, iNKT cells act as a link between the innate and the adaptive immune system, and they are able to produce the highest amounts of Th1, Th2, Th17, or regulatory cytokines upon activation [51,52,53,54]. Moreover, there is evidence of communication between cellular metabolic and immune signaling pathways for proper iNKT cell development and function. Thus, functionally, these cells present an anti-inflammatory or prohomeostatic role in disease development or inflammatory activity, with relevant roles in host defense [54].

Regarding homeostasis, Yarosz et al. recently demonstrated that the Kelch-like ECH-associated protein 1 (Keap1) and nuclear factor (erythroid-derived 2)-like 2 (Nrf2) system are critical to the development and homeostasis of NKT cells [55]. Moreover, although the function of autophagy in peripheral iNKT cell homeostasis is unknown, this inhibits mitochondrial metabolism during iNKT cell development [55]. Furthermore, regarding iNKT cell immunometabolism, a role was revealed for very-long-acyl-chain sphingolipids in iNKT cell maturation in the thymus and liver homeostasis in an animal model [56].

### 2.2. Reprogramming Metabolism after Proinflammatory Stimulation

**Figure 2:**
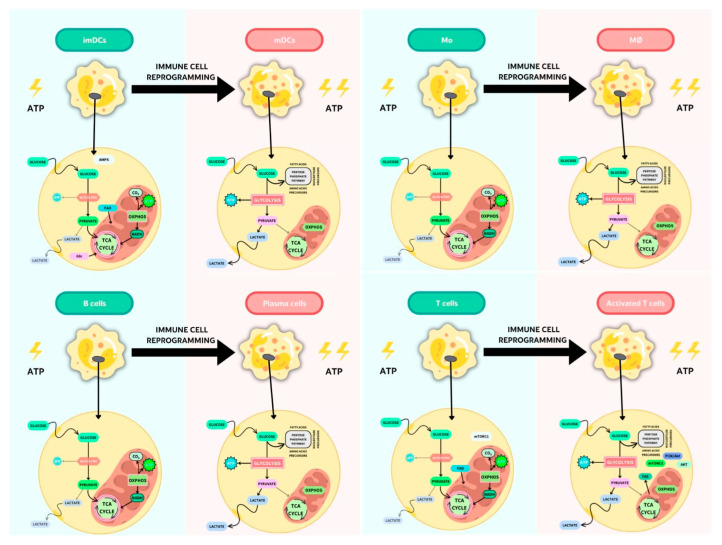
Key metabolic pathways in resting or activated APCs and T lymphocytes.

Upon inflammatory immune activation, the rapid increase in glucose consumption rates at the expense of OXPHOS brings about the generation of energy and biomass and results in the accumulation of metabolic intermediates, such as succinate, itaconate, and fumarate, which may act as immunometabolites that modulate the intracellular immune response [30] (Figure 2).

#### 2.2.1. Metabolic Changes in APCs after Proinflammatory Stimulation

DC metabolism and its effect on the efficacy of immune responses may help in the design of immunotherapeutic strategies.

It has been well established that the activation of DCs leads to increased glucose uptake and lactate production. Moreover, after a danger signal, such as PAMPs and/or DAMPs, energy in the form of ATP is generated by OXPHOS; therefore, lactate production does not reflect a commitment to the Warburg effect [14,57,58,59]. Moreover, the need for citrate by activated DCs for fueling fatty acid synthase (FAS), which is involved in endoplasmic reticulum (ER) and Golgi apparatus turnover, is met via glycolysis [59]. This glycolytic metabolism is dependent on the activation of hypoxia-inducible factor 1 (HIF-1α), an oxygen-sensitive transcription factor, and PI3K/Akt pathways, and it indicates a possible role for mTOR downstream of PI3K/Akt [58,60], whereas the induction of the OXPHOS mediator AMPK antagonizes the glycolytic pathway, inhibiting DC maturation [61]. In this sense, under certain environmental conditions, such as adequate amounts of the cytokine IL-10 or transforming growth factor (TGF)-β and contact with Treg cells or immunosuppressive drugs, DCs can become phenotypic and functionally tolerogenic [62]. Thus, tolerogenic DCs are predominantly catabolic and rely on OXPHOS and FAO for ATP production, with low glycolytic potential; a shift in the redox state, regulated via AMPK/PGC1a; and high plasticity for metabolic adaptation [32,38,63].

In contrast, immunogenic DCs exhibit anabolic metabolism and a rapid induction (mainly glycolytic) under aerobic conditions; they are an integral component of TLR signaling. Following the rapid loss of mitochondrial OXPHOS and reduced FAO [38], the TANK-binding kinase 1 (TBK1), an inhibitor of nuclear factor kappa-B kinase subunit epsilon (IKKɛ), and Akt kinase are essential for engaging the mitochondrial glycolytic enzyme hexokinase (HK)-II [64]. Moreover, an important role has been proposed for the AMPK–PPARγ co-activator 1α (PGC1α) axis in antagonizing metabolic pathways that promote DC activation, which may point to the intriguing possibility that the immunogenicity or tolerogenicity of DCs is determined by the balance between anabolic versus catabolic metabolic pathways [38,59]. In this sense, increased lactic acid levels induce a tolerogenic reprogramming during DC differentiation from monocytes [59]. Semimature DCs have been proposed as a major component in immune homeostasis [65]. These cells were first characterized by partial activation, resulting in higher levels of the expression of MHC class I and II and co-stimulatory molecules, along with lymphatic-node-homing capacity but a lack of inflammatory cytokine secretion [66]; however, this concept seems to be more complex, and other factors are implicated [67,68]. In this sense, Nguyen-Phuong et al. recently showed that blocking acetyl-CoA carboxylases 1 and 2 (ACC1/2), isozymes that regulate fatty acid metabolism (FAS in the cytoplasm and FAO in the mitochondria), in DCs favors the FAO pathway. Furthermore, they reported that the ACC1/2 blockade in DCs in a proinflammatory setting induces a semi-maturation phenotype [35].

When monocytes are studied, following proinflammatory or anti-inflammatory signals, these APCs differentiate into proinflammatory macrophages (M1) or anti-inflammatory macrophages (M2), respectively, at the affected tissue. This M1–M2 polarization is a plastic and dynamic process that is tissue specific. This implies an active characteristic metabolic state: while M1 macrophages primarily use glucose consumption and lactate excretion, M2 macrophages use OXPHOS, FAO, and mitochondrial respiration [69]. Thus, Rosa et al. proposed that FAO induces M2 [11,70]. Moreover, the repolarization of M2 to M1 after infection conditions with the consequent metabolic reprogramming leads to an impaired TCA cycle in M1 and succinate accumulation [30].

In the case of B cells, following activation through the BCR or PAMP/DAMP receptors, these cells induce their proliferation and differentiation into antibody-secreting plasma cells, as well as regulatory and memory B lymphocytes. Upon either LPS or BCR stimulation, B cells increase oxygen consumption and cause a marked upregulation in glucose; concretely, B lymphocytes have a balanced increase in lactate production and oxygen consumption following this activation, with proportionally increased glucose transporter GLUT1 expression, mitochondrial mass, and amino acid transport [44,71]. Thus, B cells rely almost exclusively on glucose metabolism to support expansion [47]. Moreover, while in T cells, increased glucose uptake is associated with aerobic glycolysis, in B cells, this glucose is used in the pentose phosphate pathway (PPP) to produce nicotinamide adenine dinucleotide phosphate (NADPH) and ribose 5-phosphate to riboneogenesis, which is fundamental for supporting antibody production [72]. Key molecular regulators that control metabolism in B cells include the PI3K signaling cascade and mTOR [72]. After activation, these cells use metabolites not only from the glycolytic and TCA cycle but also in anabolic pathways to generate FAs. These de novo synthesized FAs are critical for the initial expansion of the ER, which conditions the activation of all machinery-associated protein production to immunoglobulin synthesis [43]. In this sense, plasma cells are the only cells that secrete high levels of antibodies throughout their lives. This production of antibodies occurs during and after infections [43]. Thus, Lam et al. (2016) proposed that this antibody secretion and plasma cell survival (and, therefore, the energy requirements) are linked. Furthermore, this group demonstrated that long-lived plasma cells after increased glucose requirements produce more immunoglobulins than short-lived plasma cells that import less glucose [73].

Currently, there is open talk of the differentiation of naive B cells into regulatory B (Breg) cells, which produce the anti-inflammatory cytokine IL-10 and complex immunometabolism [72,74,75]. In this line, Menon et al. proposed that the IFN-α levels produced by plasmacytoid DCs induce the differentiation into Breg cells that restrains inflammation in autoimmune diseases [76]. Moreover, Jiang et al. recently suggested that defects in the number and function of specific Breg cells disturb immunologic homeostasis and can contribute to autoimmune disease development [77]. Thus, Rosser et al. summarized microenvironmental stimuli that induce Breg cell differentiation and the role of immunometabolism in Breg cell function, showing that Breg cell activation is poorly characterized in bioenergetic terms and depends in part on glycolysis. Moreover, they proposed that low oxygen levels in cancerous or inflammatory tissues could induce Breg cell differentiation [72]. Indeed, in this development, the intracellular cholesterol metabolism and signaling pathways to produce IL-10 are interconnected [78].

#### 2.2.2. Metabolic Changes in T Cells after Proinflammatory Stimulation

Metabolic reprogramming of T cells upon stimulation contrasts with B cell reprogramming, because T lymphocytes pass from a lower glycolytic flux when resting to a higher one, thus enhancing this pathway. Instead, in B cells, tolerance greatly affects B cell metabolic reprogramming [44]. For this, the immunometabolism of T cells presents interesting therapeutic potential.

Full activation of T lymphocytes occurs in lymphatic nodes, where naive T cells interact with mature peptide-carrying DCs via MHC class I or II molecules: TCR involvement (signal 1), in the context of co-stimulation (signal 2), and the production of effector cytokines (signal 3). However, for an absent or incomplete signal 2, anergic T cells would emerge. This implies a mechanism for the maintenance of T cell anergy, with failure to upregulate the metabolic machinery [16].

Thus, after full activation, resting naive T cells proliferate and differentiate toward T effector cells. In this process, the first two daughter T cells display phenotypic and functional indicators of being differentially fated toward effector and memory lineages [79], whereby metabolic pathways cooperate with transcription programs to maintain differential cell fates following asymmetric T cell division [80]. Thus, activated CD4^+^ T lymphocytes can differentiate into a Th response designed to fight bacterial or fungal antigens, while activated CD8^+^ T cells can differentiate into cytotoxic T cells to combat viral infections.

Memory T cells present an oxidative metabolism [81,82], governed by transcription factor c-myc [83]. Regarding subpopulations, memory CD4^+^ T cells and regulatory T cells perform FAO for their survival and metabolic needs. Moreover, it was demonstrated that lysine acetylation of glyceraldehyde 3-phosphate dehydrogenase in CD8^+^ T cells in the presence of short-chain FAs (SCFAs), such as acetate, increases glycolysis-promoting naive CD8^+^ T-cell differentiation into memory T cells [84].

Resting naive T cells after differentiation into effector T cells balance metabolic pathways from catabolic metabolism to anabolic energy metabolism. This is driven predominantly by the glycolytic–lipogenic pathway and is associated with glutamine oxidation that fuels mitochondrial OXPHOS through the TCA cycle; it is regulated via the mTOR-dependent nutrient-sensing pathway stimulated downstream of PI3K/Akt pathways [46]. Moreover, mTORC1 signaling is essential for T cell differentiation into effector CD4^+^ Th1 and Th17 cells, as well as cytotoxic CD8^+^ T cells [27,81] versus CD4^+^ Th2 cells, which display high levels of mTORC2 activity [82]. Moreover, AMPK stimulates catabolic pathways, with autophagy providing energy for CD4^+^ Th1 and Th17 cell proliferation in an inflammatory state, such as infections [26]. AMPK helps T cell differentiation and Treg cell function, supports T cell survival in glucose-limited conditions, supports T cell quiescence via FAO, and stimulates FAO in memory CD8^+^ T cells. Furthermore, Th17 cells mainly perform glycolysis thanks to the fact that they possess HIF-1α, which is an oxygen-sensitive transcription factor that regulates glycolytic gene expression. However, Treg cells perform glycolysis, FAO, and OXPHOS. Proinflammatory CD4^+^ T cells rely on glycolysis. Thus, similar to other leukocytes, such as APCs, Th17 cells use glycolysis for effector inflammatory function; however, when this metabolic pathway is blocked, T cells become Treg cells [85]. In a recent study, McTernan et al. demonstrated through in vitro assay that ethanol alters naive T cell metabolism with an increase in glycolysis and impaired OXPHOS, which disrupts mitochondrial repair processes and promotes Th1 CD4^+^ T lymphocytes [86]. Jones et al. showed the main metabolic pathways for different functions in human CD4^+^ and CD8^+^ T cells. Thus, increased expression of HK-II with higher basal glycolysis was demonstrated in CD4^+^ T cells. However, cytokine production in CD8^+^ T cells is more reliant on OXPHOS [83].

Treg cell metabolism in vivo is dependent on the environment and type of immune response orchestrated. Thus, Treg cells that develop in vivo present a resemblance to effector T cells in that they depend on glycolysis-driven lipogenesis, with the raptor/mTORC1 pathway that promotes cholesterol and lipid metabolism, for their proliferation and function [46,87]. In this sense, it has been reported that inhibition of the mTOR pathway with rapamycin can balance the response between regulatory and effector T cells [72]. Moreover, it was proposed that Treg cells perform FAO for energy and survival, similar to memory CD4^+^ T cells [54].

T cells are highly influenced by nutrient uptake from their environment, and changes in the overall nutritional status, such as malnutrition or obesity, can result in altered T cell metabolism and behavior. In states of severe malnutrition or starvation, T cell survival, proliferation, and inflammatory cytokine production all decrease, as do T cell glucose uptake and metabolism. The altered T cell function and metabolism seen in malnutrition are associated with altered adipokine levels, most particularly decreased leptin [88].

### 2.3. Modulation of Immunometabolic Pathways in Obesity and Type 2 Diabetes as an Intervention Tool

Immunometabolic pathways in patients with obesity and/or T2DM are altered. Diabetes is essential for immunometabolism studies, since high glucose levels over time trigger processes such as endothelial inflammation, increased mitochondrial oxidative stress, and decreased nitric oxide (NO), producing systemic alterations related to immunity. T2DM, the most common form of diabetes (∼90%), presents a systemic inflammatory response coupled with insulin resistance (IR) or decreased metabolic response to insulin in various tissues, such as adipose tissue, liver, and skeletal muscle, as well as by reduced insulin synthesis in the islets of Langerhans [89,90]. Thus, after the APC–T lymphocyte interaction and activation process, this process is favored by factors such as insulin. Moreover, insulin resistance (IR) and a change in receptors affect T cell function, specifically polyclonal CD4 cell activation and effector cytokine production, such as Th1 and Th17 cells. Thus, IL-17 production affects IR signaling in macrophages, altering its activation.

Regarding B cells, an increase in IgG production is related to IR [91]. In addition, it has been shown that B lymphocytes secrete proinflammatory cytokines, including IL-8, along with a decrease in IL-10 production, compared to B cells from subjects without diabetes.

During the progression of diabetes, hyperglycemia promotes mitochondrial dysfunction and induces the formation of reactive oxygen species (ROS) that cause oxidative stress in several tissues, such as blood vessels and pancreatic beta cells [24].

Surendar et al. showed that a combination of factors, such as decreased adiponectin and increased Th1 and Th17 cell glycolysis, can favor IR, contributing to obesity [92]. Similarly, Damas et al. demonstrated that Th17 cells and macrophages accumulated in visceral adipose tissue contribute to altered glycemic status in obese subjects [93].

#### Modulation of Aerobic Glycolysis and TCA with Traditional Drugs

In obesity and diabetes, the proinflammatory microenvironment alters immunometabolism, favoring the aerobic glycolysis pathway in both APCs and T cells, albeit with signatures specific to each cell type. In contrast, OXPHOS generally favors an anti-inflammatory phenotype, such as that of tolerogenic DCs, M2, and Treg cells. Thanks to the knowledge of the energy production pathways involved in the processes of activation, proliferation, and survival of immune cells, different targeted therapies are applied that act on the immunometabolism of the cells affected in said pathologies. For this, therapeutic immunomodulators described, such as dimethyl fumarate (DMF), metformin, methotrexate, and rapamycin, are used to induce metabolic reprogramming with anti-inflammatory effects [31].

### 2.4. Modulation of Metabolic Pathways as a Therapeutic Strategy: The Nutraceutical Approach

An approach in therapies to modulate the exacerbated proinflammatory response in T2DM and obesity could be the use of nutraceuticals as an adjuvant to redirect the alterations in the metabolic pathways that occur in immune cells in a proinflammatory microenvironment. The propose is the regulation of the inflammation associated with these pathologies; thus, intervention in metabolic pathways through the modulation of metabolic energy with nutraceuticals alone or combined with traditional drugs is an attractive strategy that allows inducing important changes in immunocellular properties in obesity and T2DM (Table 1).

#### 2.4.1. Nutraceutical Anti-Inflammatory and Antioxidation Functions

**Figure 3:**
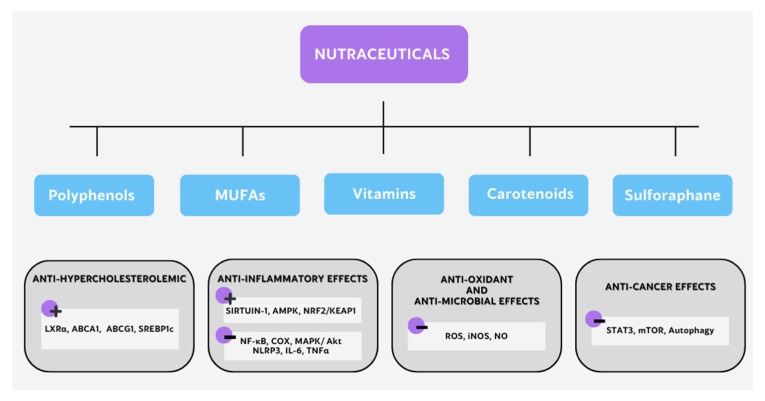
Different mechanisms of action of nutraceuticals with immunometabolic potential.

Nutraceuticals have demonstrated a wide range of health effects, such as anti-inflammatory, anticancer, antioxidant, and prebiotic effects and their regulation of lipid metabolism [141]. Next, we focus on those nutraceuticals that have shown immunometabolic abilities (Figure 3).

#### 2.4.2. Polyphenols

These compounds are phenylpropanoids of plants origin that are related to a decrease in cardiovascular risk, capable of showing anti-inflammatory, anti-hypertensive, anti-platelet, and antioxidant function [141,142]. The antioxidant action (ROS scavenging, oxidative stress protection, thiol redox stabilization, and membrane lipid peroxidation attenuation) or pro-oxidant activity (ROS production, thiol redox alteration, membrane lipid peroxidation, and oxidative stress) of polyphenols is able to regulate epigenetic factors by oxidant and thiol-redox-mediated signaling modulation. For example, our group demonstrated that naringenin regulates the liver X receptor (LXR)α expression in macrophages by modulating AMPK [94]. Wang et al. showed that naringenin induces the aryl hydrocarbon receptor (AhR)-mediated signaling pathway in Treg cells [95]. Moreover, Li et al. demonstrated that naringenin improves insulin sensitivity in gestational diabetes mellitus through AMPK in nonimmune cells (e.g., skeletal muscles) in mice [143].

Resveratrol is a plant-derived polyphenol with pleiotropic biological properties, a potent antioxidant nutraceutical, and a SIRTUIN-1 activator, which is able to partially inhibit the enhanced IL-6 production after β-glucan stimulation in monocytes [96]. Svajger et al. showed that resveratrol induces DC-associated tolerance, suggesting that these effects may be associated with molecular targets through nuclear factor kappa-light-chain-enhancer of activated B cells (NF-κB) translocation [62]. Moreover, Shabani et al. demonstrated that resveratrol treatment can decrease M1 and increase Treg cell infiltration into skeletal muscle in mice fed a high-fat diet (HFD). Moreover, they observed that resveratrol decreases inflammation in skeletal muscle with an AMPK expression increase and a p38 mitogen-activated protein kinase (MAPK) and c-Jun *N*-terminal kinase (JNK) decrease [97]. Thus, their results showed the metabolic reprogramming toward M2 and Treg cells is balanced after resveratrol treatment in HFD mice.

Furthermore, moderate doses of resveratrol promote OXPHOS and mitochondrial biosynthesis in DCs from mice and humans via activating SIRTUIN-1 and AMPK [38,144], as well as via augmenting PGC1α expression to prevent DC maturation and immunogenic activation [38].

Quan et al. demonstrated that resveratrol suppresses the reprogramming of macrophages into an endotoxin-tolerant state through the activation of AMPK [98]. In line with this, Chan et al. showed that this polyphenol blocks iNOS expression and NO generation in these cells [99]. Malaguarnera summarized that resveratrol decreases NF-κB activation and COX on activated macrophages and attenuates the TLR4–TRAF6, MAPK, and AKT pathways [100]. Thus, resveratrol attenuates monocyte-to-macrophage differentiation via GSH upregulation through AMPK activation [101].

Moreover, resveratrol’s capacity to act on T cell differentiation to decrease the inflammatory-associated response was analyzed. This polyphenol can inhibit inflammation through the ratio between Th17 cells and Treg cells and the Th1 cell/Th2 cell balance; acting as a SIRTUIN-1 agonist, it can deacetylate the transcription factor STAT3 and alter nuclear factors essential to the process of lymphocyte differentiation [102].

Curcumin is an important anti-inflammatory and antioxidant compound playing a key role in many cellular processes, including inhibition of STAT3 activation in nonimmune cells, such as adipocytes and trophoblast cells; inhibition of NF-kB activation in pregnancy complications; and activation of the NRF2/KEAP1 pathway [145,146,147,148]. Campbell et al. demonstrated that polyphenols, such as carnosol and curcumin, can decrease glycolysis and spare respiratory capacity in LPS-induced DC stimulation, via AMPK activation and mTOR signaling inhibition. Thus, they suggested that polyphenol supplementation may be useful to regulate inflammation through immunometabolism in metabolic disease [103]. Furthermore, we described that curcumin induces AMPK phosphorylation and increases LXRα mRNA and protein expression. Curcumin upregulates the expression of genes involved in cholesterol transport and metabolism, such as ATP-binding cassette (ABC) transporters ABCA1 and ABCG1, as well as sterol response element-binding protein 1c (SREBP1c), showing a possible mechanism for understanding the hypocholesterolemic effects of curcumin and expanding the knowledge about LXRα regulation by AMPK [104]. Piperine blocks ABCA1 degradation and increases cholesterol efflux in macrophages [149]. Furthermore, Liu et al. demonstrated that piperine can inhibit the M1 polarization of macrophages through downregulation of proinflammatory cytokine expression and a decrease in CD11c and Gal-3 M1-like polarization markers. Reprogramming toward M2 cannot be achieved in the adipose tissue of obese mice but may decrease insulin resistance [106]. However, controversial results have been proposed for piperine. Pan et al. and He et al. showed that this nutraceutical increases mTORC1 activity in resident peritoneal M1 to produce IL-6 and TNF-α, thus boosting their functions against bacterial infection [107,108].

Moreover, the effects of curcuminoids and piperine are greater when combined than when alone in reducing serum malondialdehyde levels but without affecting TNF-α, leptin, or adiponectin in blood [4,105]. Although the synergistic effect of curcumin and piperine on global metabolism has been well established, no studies have been conducted on immunometabolism. More conducive studies are needed to clarify the power of these combined nutraceuticals.

Quercetin, a common polyphenol in nature, is an aglycone bioflavonoid widely used for the treatment of metabolic and inflammatory disorders [150]. Huang et al. demonstrated that quercetin can inhibit LPS-induced DC maturation through decreased surface expression of MHC class II and co-stimulatory molecules and a reduction in proinflammatory cytokines/chemokines. Moreover, this nutraceutical can abrogate the ability of LPS-stimulated DCs to induce Ag-specific T cell activation, both in vitro and in vivo. Furthermore, quercetin may reduce MAPK, Akt, and NF-κB signaling pathway activation and diminish Ag-specific T cell activation [109]. Similar to what is observed for resveratrol, quercetin reduces macrophage NO production through scavenging of NO and a reduction in iNOS gene expression [99]. Furthermore, quercetin reduces specific immunoglobulin (Ig) E production in plasma cells. In this sense, this polyphenol can modulate the Th2 response through IL-4 and IL-5 cytokine reduction [110]. Moreover, Yang et al. determined that quercetin supplementation in the diet in an animal model can increase IgA and IgM in serum. They demonstrated that quercetin also increases IL-4, complement component 4 (C4), and TNFα production through NF-κB signaling pathway activation [151].

Baicalin is a major flavonoid glycoside present in the dry roots of Scutellaria baicalensis and is used to treat hypertension [111]. Thus, this nutraceutical can act through TLR4, BCR, and TCRαβ, present in APCs and T cells [152], to regulate immunometabolism in the host. Furthermore, baicalin can induce ABCA1 and ABCG1 cholesterol transporters. In this sense, it has been demonstrated that baicalin increases PPARγ and LXRα protein levels in macrophages [111].

Berberine, a phytoalkaloid, presents hypoglycemic effects on T2DM [153]. Thus, Reddi et al. showed the anti-inflammatory potential of this nutraceutical via decreasing NF-κB in activated monocytes [112], and Gong et al. observed its inhibitory effects on M1 polarization through interfering with TLR4 interaction and disturbing the TLR4/MyD88/NF-κB signaling pathway [113]. Daien et al. demonstrated that dietary fiber supplementation in healthy individuals is associated with increased B10 cells in peripheral blood [123]. Moreover, short-chain fatty acids (SCFAs) from high dietary fiber and microbiota metabolism can bind to specific G-protein-coupled receptors (GPCR) of immune cells, thereby changing their phenotype. These metabolites can also directly fuel specific metabolic pathways to affect immune function [154].

Dietary nutraceuticals, such as SCFAs, can act as tolerogenic modulators in immunometabolic responses. Thus, acetate, propionate, and butyrate, which are mainly derived from the microbiota metabolism, promote a tolerogenic response induced through B and Treg cells via epigenetic mechanisms; for example, butyrate inhibits histone deacetylase that increases FOXP3 expression and enhances acetylation at histone H3 in the FOXP3 promoter [120,121]. This protective mechanism delays the onset of diabetes [119]. Moreover, it has been demonstrated that SCFAs inhibit the production of proinflammatory cytokines and chemokines, as well as the recruitment of leukocytes to injured tissue [155]. The anti-inflammatory activity of SCFAs has been described in intestinal barrier function, showing that acetate, propionate, and butyrate stimulate the formation of tight junctions through inhibition of the NLRP3 inflammasome and autophagy in the intestinal barrier [156].

Moreover, it was recently demonstrated that SCFAs promote double-negative T cell differentiation in intestinal microenvironmental immunity through OX40 via inhibition of the NLRP3 inflammasome [122].

Daïen et al. recently demonstrated that acetate promotes IL-10-producing Breg or B10 cells [123]. Moreover, it has been shown that this molecule may increase glycolysis-promoting naive CD8+ T cell differentiation into memory T cells [84].

Monounsaturated fatty acids (MUFAs) are chemically classified as FAs that present one double bond in the carbon chain. There are three main classes of MUFAs: omega-3 omega-6, and omega-9 (*n*-3, *n*-6, and *n*-9, respectively). They can act as antioxidants by modulating the antioxidant signaling pathway and may regulate inflammatory processes [157]. Thus, Dangardt et al. demonstrated that *n*-3 MUFA supplementation increases serum levels and decreases TNFα, IL-6, and IL-1β levels in the PBMCs of subjects with obesity [114]. In line with this, Zhao et al. revealed that the treatment of people with obesity with *n*-3 MUFAs, such as linolenic acid, decreases free plasma FAs, IL-6, and TNFα levels and increase PPARγ expression in mononuclear cells (PBMCs) [11,115]. Furthermore, oleic acid (OA) is an *n*-9 MUFA, recognized as a versatile nutraceutical and effective biomolecule, with potent antioxidant capacity because it can directly regulate both the synthesis and the activity of antioxidants enzymes [158]. Thus, Camell et al. described that dietary OA induces M2 in the mesenteric adipose tissue of mice [159]. In line with this, Charlett et al. published that OA decreases COX-2, TNFα, IL-6, and IL-12 expression in LPS-stimulated M1, showing anti-inflammatory and antifungal properties [116]. Hou et al. reported that OA supplementation increases the AMP/ATP ratio and AMPK activation, as well as inhibiting the NF-κB pathway in M1 [117]. Moreover, Hong et al. proved that OA treatments have anti-inflammatory effects through inhibiting proinflammatory mediators, including PI3K, Akt, MAPKs, NF-κB, NOS2, and COX2 in M1 [118]. Regarding T cells, Gorjão et al. demonstrated that OA stimulates T lymphocytes, inducing their proliferation, while other saturated or *n*-3 fatty acids decrease it [160].

Vitamins are essential micronutrients that are synthesized by bacteria, yeasts, and plants but not by mammals [161]. They play key roles in inflammation and in basic metabolic pathways [162].

Vitamin B1 is an essential cofactor in the maintenance of the TCA cycle. Thus, Kunisawa et al. showed that naive B cells in Peyer’s patches use the vitamin-B1-dependent TCA cycle for the generation of ATP. However, IgA-producing plasma cells switch to using glycolysis for the generation of ATP and shift to a catabolic pathway for IgA secretion. They demonstrated that a vitamin-B1-deficient diet in mice prevented intestinal lamina propria B cell differentiation after proinflammatory stimuli [124].

Vitamin D is available in two distinct forms: ergocalciferol (vitamin D2) and cholecalciferol (vitamin D3) or 1.25(OH)2D3. Vitamin D3 is the main form of vitamin D in the diet of most people and is the form that is synthesized in the skin. Vanherwegen et al. summarized that 1.25(OH)2D3 interferes with the expression of genes involved in processes such as glycolysis, OXPHOS, and TCA, as well as genes in the PI3K/Akt/mTOR signaling pathway in DCs [126]. Thus, vitamin D3 induces glycolytic enzyme 6-phosphofructo-2-kinase/fructose-2,6-biphosphatase 4 (PFKFB4) expression, leading to metabolic reprogramming toward aerobic glycolysis and glucose oxidation in tolerogenic DCs to induce functional Treg cells [127]. Moreover, other studies published have reflected that vitamin D and/or resveratrol can induce tolerance in monocyte-derived DCs [62,125].

Related to macrophages, vitamin D3 acts as a stimulator of NO production in the human HL-60 cell line. This occurs by increasing iNOS gene expression, which can lead to the suppression of *Mycobacterium tuberculossus* infection [126]. This antimicrobial activity is associated with an increase in ROS levels mediated by NADPH oxidase and PI3K [128]. In this sense, Vanherwegen et al. concluded that vitamin D3 can increase glycolytic immunometabolism typical of the M1 phenotype [126].

Moreover, vitamin D3 presents anti-inflammatory activity in monocytes/macrophages by decreasing both the protein and the mRNA levels of TLR-2 and TLR-4.

This reduces IL-6 and TNF-α levels [129].

Salomon et al. showed the connection between myeloid lipid metabolism and vitamin D3 receptor signaling in the context of *M. tuberculosis* infection. Thus, they demonstrated that vitamin D3 regulates pro-adipogenic PPARy in activated macrophages, leading to the inhibition of lipid droplet induction by this nutraceutical [130]. Moreover, it has been published that vitamin D enhances the IL-10 expression of activated B cells [131], which can lead to B10 cells. In line with this, low-dose combined vitamin D3/dexamethasone promoted IL-10 production in DCs and B cells from dyslipidemic mice [132]. Moreover, this combined treatment decreased the percentage of IFNγ-producing CD4 and CD8 T cells in dyslipidemic mice, leading to tolerogenic immunometabolism with a decrease in the Th1 pattern response [132]. Thus, this evidence highlights vitamin D3 as an inducer of metabolic changes in immune cells.

Vitamin C (also known as ascorbic acid) is a potent antioxidant nutraceutical. Chen et al. demonstrated that this molecule may inhibit LPS-induced ROS, DNA damage, TNF-𝛼, IL-6, and p38 MAPK in the macrophages of patients with community-acquired pneumonia. Moreover, vitamin C inhibited autophagy in these LPS-induced macrophages [133]. In this sense, Morante-Palacios et al. published that vitamin C deeply enhances DNA demethylation during monocyte-to-DC differentiation and later maturation. Moreover, they demonstrated that this nutraceutical triggers extensive demethylation at NF-κB/p65-binding sites, together with concordant upregulation of antigen presentation and immune-response-related genes during DC maturation [134]. Furthermore, Tan et al. showed that vitamins C and E inhibit the NF-κB signaling pathway in DCs. This, together with the oxidative pathway blocked in vitamin-C-/vitamin-E-treated DCs, produced Treg-cell-mediated responses [135].

Vitamin E (tocopherol) plays an important role as a potent lipid-soluble antioxidant agent in immune cells, which is found in higher concentrations, being one of the most effective nutraceuticals to modulate immunometabolism [163]. Moreover, this nutraceutical vitamin E plays an important anticancer activity, modulating the NRF2/KEAP1 pathway [148]. Vitamin E presents a protective role, acting against oxidation of membrane PUFAs, due to their high metabolic activity and their defensive function [163]. Regarding DCs, as already mentioned before, Tan et al. studied the antioxidant function of vitamin E alone or combined with vitamin C, showing that lower doses of α-tocopherol increase HLA-DR, CD40, CD80, and CD86 membrane expression [135], thus improving DC phenotypical maturation. Moreover, Xuan et al. proved that higher doses of α-tocopherol inhibit NF-κB-mediated DC functional maturation [136]. Thus, opposite effects are observed depending on the concentration of vitamin E used. In addition to these studies, it can be deduced that low doses of vitamin E combined with higher doses of vitamin C do protect DCs from their phenotypical and functional activation. Moriguchi et al. demonstrated that vitamin E supplementation in elderly mice reduces inflammatory cytokine production and improvement in T cell proliferation and improves alveolar macrophage phagocytic activity [137]. Marko et al. showed that vitamin E supplementation in elderly mice and humans may enhance early events in T cell activation, including the interaction between naive CD4 T cells and APCs [138].

Carotenoids represent 40-carbon molecules found in red, yellow, and orange fruits and vegetables but also in some animal products (i.e., eggs and fish), which are widely distributed in nature. They have diverse biological functions, acting as antioxidant and anti-inflammatory agents [164]. Thus, lycopene and astaxanthin are strong antioxidants that decrease the risk of both cancer and cardiovascular diseases [139]. Astaxanthin can reduce heart inflammation and balance the blood levels of LDL-C and HDL-C, contributing to a decrease in macrophage infiltration and apoptosis in vascular lesions [139,165]. Astaxanthin may block oxLDL production and uptake by activated intravascular macrophages to inhibit the release of ROS, NO, and proinflammatory cytokines in injured tissue [139,166].

Sulforaphane (1-isothiocyanate-4-methyl-sulfinylbutane, SFN) is present in cruciferous vegetables, such as broccoli and cauliflower [167,168]. This nutraceutical shows a powerful anti-inflammatory effect [168]. Furthermore, this molecule is an important antioxidant and anticancer agent, preventing cancer occurrence and improving the chemotherapy response [148,169]. Regarding immunometabolism, SFN may inhibit the release of NO, COX-2, iNOS, TNF-α, IL-6, and IL-1β in LPS-stimulated macrophages [170]. Recently, Bahiraii et al. demonstrated that SFN decreases M1 marker expression, such as IL-1β, IL-6, TNF-α, iNOS, NO, and ROS. Moreover, they showed that this nutraceutical blocks pyruvate kinase M2 (PKM2) in M1 macrophages [140].

#### 2.4.3. Delivery Systems for Nutraceuticals

The usage of nutraceuticals to prevent and treat obesity and/or T2DM is limited by several features, such as poor water solubility, low bioavailability, uncontrolled release, difficulty in crossing biological barriers due to low permeability, and low efficacy. One of the strategies in biomedicine to overcome these disadvantages is the use of nanostructures, such as nanocarriers. Thus, Hoti et al. summarized nanotechnology-based delivery systems used for nutraceutical transportation [171]. Moreover, this group contextualized modified cyclodextrin (CD)-based nanosponges (NSs; CD-NSs) as efficient encapsulating agents for delivering nutraceuticals with controlled kinetics through the topical, oral, and parenteral routes. CD-NSs are the most preferred, advanced, biocompatible, and natural systems to deliver nutraceuticals. These nanocarriers are used as delivery systems of several nutraceuticals, such as quercetin, curcumin, and resveratrol [171]. Soni et al. demonstrated that SFN-loaded nanostructured lipid carriers (NLCs) improve oral efficacy against cancer. The optimized SFN-loaded NLC formulation Precirol^®^, with ATO 5 (solid lipid) and vitamin E (liquid lipid) as the lipid phase, represents a great strategy for the improved efficacy of SFN after oral administration [172]. Although efficient encapsulating agents for delivering nutraceuticals are being developed [171,172], few studies to date have shown its impact on immunometabolism. In this sense, Osali demonstrated that nanostructures functionalized with curcumin decrease the concentration of malondialdehyde. Furthermore, this nano-curcumin increased brain-derived neurotrophic factor and IL-10 levels, as well as antioxidant capacity, in the blood from subjects with metabolic syndrome [173].

## 3. Conclusions

We conclude that nutraceuticals are immunometabolic modulators with an anti-inflammatory function, which are able to decrease the activated inflammatory metabolic state of APCs and T lymphocytes in obesity and/or T2DM pathologies, promoting tolerogenic metabolic reprogramming in these immune cells. Although there have been important discoveries in basic research, further effort is necessary to confirm the immunometabolic properties demonstrated by nutraceuticals in translational immunology, such as clinical assays.

## Figures and Tables

**Table 1:** Nutraceuticals with a modulator effect on immunometabolism.

Nutraceuticals	Effects/Immunometabolic Signaling Pathways		References
	APCs	T Lymphocytes	
**Naringenin**	Anti-inflammatory effects/↑LXRα/↑AMPK (MΦ)	-	[94]
	-	↑AhR in Treg cells	[95]
**Resveratrol**	Anti-inflammatory effects/↑SIRTUIN-1/↓IL-6 (Mo)	-	[96]
	Tolerogenic DCs/↓NF-κB	-	[62]
	↓M1, ↑M2/↑AMPK, ↓p38 MAPK, ↓JNK	↑Treg cells	[97]
	Tolerogenic DCs/↑OXPHOS, mitochondrial biosynthesis, ↑SIRTUIN 1, AMPK (DCs)	-	[38]
	Tolerogenic DCs/↑PGC1α	-	[38]
	↓M1/↑AMPK	-	[98]
	↓M1/↓iNOS, ↓NO	-	[99]
	↓M1/↓NF-κB, ↓COX, ↓TLR4-TRAF6, ↓p38 MAPK, ↓Akt	-	[100]
	↓Mo-to-M1 differentiation/↑GSH, ↑AMPK	-	[101]
	-	↓Th17 cells/↓Treg cells, ↓Th1 cells/↓Th2 cells/↓STAT3	[102]
**Carnosol**	↓Glycolysis, ↓mitochondrial respiration/↑AMPK, ↓mTOR (mDCs)	-	[103]
**Curcumin**	↓Glycolysis, ↓mitochondrial respiration/↑AMPK, ↓mTOR (mDCs)	-	[103]
	↑Lipid metabolism/↑AMPK, ↑LXRα, ↑ABCA1, ↑ABCG1, ↑SREBP1c (M1)	-	[104]
**Piperine**	↑Cholesterol efflux/↑ABCA1 (M1)	-	[105]
	↓M1 polarization/↓ inflammatory cytokines, ↓CD11c, Gal-3 (M1)	-	[106]
	↑IL-6, TNF-α/↑mTORC1 (M1)	-	[107,108]
**Quercetin**	↓DC maturation/↓MAPK, ↓Akt, ↓NF-κB	↓Ag-specific T cell activation	[109]
	↓M1/↓iNOS, ↓NO	-	[99]
	↓Th2 response/↓specific IgE (plasma cells)	↓Th2 response/↓IL-4, ↓IL-5	[110]
**Baicalin**	↑Lipid metabolism/↑ABCA1, ↑ABCG1; ↑PPARγ, ↑LXRα (M1)	-	[111]
**Berberine**	Anti-inflammatory role/↓NF-κB (Mo)	-	[112]
	↓M1 polarization/↓TLR4/MyD88/NF-κB	-	[113]
**MUFAs**	Anti-inflammatory effects/↓TNFα, ↓IL-6, ↓IL-1β (Mo)	Anti-inflammatory effects/↓TNFα, ↓IL-6, ↓IL-1β (lymphocytes)	[11,114,115]
	Anti-inflammatory effects/↓COX-2, ↓TNFα, ↓IL-6, ↓IL-12 (M1)	-	[116]
	↑AMP/ATP ratio, ↑AMPK, ↓NF-κB pathway (M1)	-	[117]
	Anti-inflammatory effects/↓PI3K, ↓Akt, ↓MAPKs, ↓NF-κB, ↓NOS2, ↓COX2 (M1)	-	[118]
**SCFAs**	↑Breg cells/Epigenetic mechanism	↑Treg cells/↓histone deacetylase, ↑FOXP3	[119,120,121]
	-	↑DN T cell differentiation/↑OX40, ↓NLRP3	[122]
	↑Breg cells/↑IL-10 (B cells)	-	[123]
**Vitamin B**	ATP generation/naive B cells vitamin B1-dependent TCA cycle	-	[124]
**Vitamin D**	↑Aerobic glycolysis, ↑OXPHOS, ↑TCA/↑PI3K/Akt/mTOR (DCs)	-	[62,125,126]
	Reprogramming aerobic glycolysis, glucose oxidation/PFKFB4 (DCs)	Reprogramming aerobic glycolysis, glucose oxidation/PFKFB4 (DCs: Treg cells)	[127]
	↑M1 with antimicrobial activity/↑iNOS, ↑NO	-	[126]
	↑M1 with antimicrobial activity/↑ROS	-	[128]
	Anti-inflammatory activity/↓TLR-2, TLR-4, ↓IL-6, ↓TNF-α (Mo/MΦ)	-	[129]
	↑Infected M1 with antimicrobial activity, lipid metabolism/↓PPARy, lipid inhibition (M1)	-	[130]
	↑Breg cells/↑IL-10 (B cells)	-	[131]
	-	↓Th1 response, ↑Treg cells/↓IFNγ-producing CD4, CD8 T cells (mice)	[132]
**Vitamin C**	Antioxidant and anti-inflammatory effects/↓ROS, ↓DNA damage, ↓TNF-𝛼, ↓IL-6, ↓p38 MAPK, ↓autophagy, ↑DNA demethylation (M1)	-	[133]
	↑DNA demethylation (DC)	-	[134]
	Vitamin C and E inhibit oxidative pathway/NF-κB (DCs)	Vitamin C and E inhibit blocks oxidative pathway/Treg-cell-mediated responses	[135]
**Vitamin E**	↓Activated-DC-/↓NF-κB-mediated DC functional maturation (mDCs)	-	[136]
	Anti-inflammatory effects/↓inflammatory cytokines	-	[137]
	-	↑Activation/↑CD4–APC interaction	[138]
**Astaxanthin**	↓FAO/↓oxLDL production, ↓ROS, ↓NO, ↓inflammatory cytokines (M1)	-	[139]
**Sulforaphane**	Anti-inflammatory effect/↓NO, ↓COX-2, ↓iNOS, ↓TNF-α, ↓IL-6, ↓ IL-1β, ↓PKM2 (M1)	-	[140]

ABC: ATP-binding cassette transporters; AhR: aryl hydrocarbon receptor; AKT: protein kinase B; AMPK: AMP-activated protein kinase; APC: antigen-presenting cell; Breg cell: regulatory B cell; COX: cyclooxygenase; DC: dendritic cell; FAO: fatty acid oxidation; Gal-3: galectin-3; GSH: glutathione; IgE: immunoglobulin E; MΦ: macrophages; iNOS: inducible nitric oxide synthase; JNK: Jun *N*-terminal kinase; LXRα: liver X receptor alpha; Mo: monocytes; MyD88: myeloid differentiation primary response 88 protein; M1: pro-inflammatory macrophages; M2: anti-inflammatory macrophages; mTOR: mammalian target of rapamycin; NF-κB: nuclear factor kappa-light-chain-enhancer of activated B cells; NO: nitric oxide; NOS2: nitric oxide synthase 2; oxLDL: oxidized low-density lipoprotein; OXPHOS: oxidative phosphorylation; PKM2: pyruvate kinase M2; PFKFB4: 6-phosphofructo-2-kinase/fructose-2,6-biphosphatase 4; PGC1α: PPARγ co-activator 1alpha; PI3K: phosphoinositide 3-kinases; PPARγ: peroxisome proliferator-activated receptor gamma; p38 MAPK: p38 mitogen-activated protein kinases; IL-1: interleukin 1, IL-6: interleukin 6; ROS: reactive oxygen species; SREBP1c: sterol response element-binding protein 1c; TCA: tricarboxylic acid; TLR-4: Toll-like receptor 4; TNF-α: tumor necrosis factor alpha; TRAF6: TNF receptor-associated factor 6; Treg cell: regulatory T cell.

## Data Availability

Not applicable.
